# Chimerolectins: Classification, structural architecture, and functional perspectives

**DOI:** 10.1002/pro.70261

**Published:** 2025-08-15

**Authors:** Vanir Reis Pinto‐Junior, Benildo Sousa Cavada, Kyria Santiago Nascimento

**Affiliations:** ^1^ Laboratory of Biologically Active Molecules, Department of Biochemistry and Molecular Biology Federal University of Ceara Fortaleza Brazil

**Keywords:** artificial lectins, biotechnological applications, chimerolectins, lectins, structural

## Abstract

Lectins are proteins or glycoproteins capable of binding specifically and reversibly to carbohydrates, a property that, in itself, gives them great functional versatility in organisms from all kingdoms of nature. A subclass of these proteins, called chimerolectins, is composed of proteins that have at least one lectin domain associated with another functional domain, such as enzymatic domains or modules involved in molecular signaling processes. The emergence of chimerolectins throughout evolution significantly expanded the functional repertoire of lectins, allowing their action to go beyond the interaction with carbohydrates and glycoconjugates. These proteins are involved in the regulation of the immune system in humans and animals, in the defense of plants against pathogens and predators, as well as in the mediation of responses to biotic and abiotic stresses. In addition, they can act as potent lethal toxins or as factors in the infection of several pathogens and are often associated with the manifestation of symptoms of diseases, which makes them therapeutic targets of great interest. Deepening the structural knowledge of these proteins has been essential for understanding their mechanisms of action, in addition to providing solid bases for biotechnological applications and for the rational development of artificial lectins with specific functions. This approach has enabled the creation of chimerolectins with potent antiviral activity, as well as the development of new therapeutic strategies aimed at inducing death in cells of different tumor lineages.

## 1 INTRODUCTION

Lectins are defined as a class of proteins, or glycoproteins, capable of reversibly binding to carbohydrates without, however, modifying their structures. They are ubiquitous proteins in nature; that is, they are present in all living beings, performing various functions (De Coninck and Van Damme 2021). The mere property of recognizing and binding to specific carbohydrates already affords lectins with multiple functionalities, such as participation in cell signaling processes, host–pathogen recognition, cell adhesion, defense against pathogens and predators, and symbiotic processes (Jeyachandran and Vibhute 2024; Kremsreiter et al. 2021; Singh and Walia 2014; Stegmann and Lepenies 2024). Furthermore, this ability confers enormous potential for biotechnological applications, including anti‐inflammatory, antiviral, antibacterial, antifungal, and antitumor activities (Boliukh et al. 2024; Del Rio et al. 2020; Fonseca et al. 2022; Konozy and Osman 2024; Naik and Kumar 2022).

Based on one key structural classification of lectins, which involves the number and presence of domains, the chimerolectin subclass stands out. These proteins have at least one carbohydrate recognition domain (CRD) associated with one or more unrelated domains with distinct biological functions. This combination has significantly expanded the functional potential of lectins in living beings, as well as the structural and functional diversity of this class of proteins (Naithani et al. 2021).

Many chimerolectins are associated with human immune defense and oxidative stress in plants. Chimeric lectins are even associated with pathogenicity mechanisms whereby microorganisms use chimerolectins for cellular recognition and induction of disease‐related symptoms (Moustafa et al. 2004; Rahimi 2020; Vishweshwaraiah et al. 2018). In addition, some act as extremely powerful toxins or interfere with cellular osmotic balance through the formation of pores (Bortolotti et al. 2023; Chen et al. 2015; Dang et al. 2017; Rai et al. 2013). For these reasons, certain natural chimerolectins have been considered promising therapeutic targets against pathogens. When used strategically, lectins demonstrate high potential for application in healthcare and agriculture (Chettri et al. 2021).

Structural analysis of these proteins is particularly relevant since it reveals how lectin domains have been explored evolutionarily in nature. Such analyses also provide support for the rational design of artificial chimerolectins for biotechnological purposes (Notova and Imberty 2023).

In this context, the present review will discuss eight subgroups of chimerolectins, highlighting their structural and functional aspects, showcasing the diversity of this protein subclass with particular emphasis on their domains. Furthermore, we will see how natural chimerolectins have inspired the creation of synthetic chimerolectins with biotechnological applications, especially in the delivery of healthcare.

## 2 SUBGROUPS OF CHIMEROLECTINS

### 2.1 C‐type lectins

Although the classification of C‐type lectins as chimerolectins is still an ambiguous point in the literature, we chose to include this family since some of these proteins not only present a carbohydrate recognition domain (CRD) but also other domains of interest with distinct functions. C‐type lectins are calcium (Ca^2+^)‐dependent proteins for recognizing carbohydrates, but they also interact with other ligands, such as lipids and proteins. Despite forming a functionally heterogeneous group, these lectins share highly conserved structural modules in their CRDs. Proteins that contain at least one C‐type domain are present in both soluble and membrane proteins. The family is subdivided into 17 groups based on phylogenetic relationships and domain architecture. To understand their physiological functions, a detailed analysis of the three‐dimensional structure and the molecular mechanisms involved in carbohydrate recognition is essential (Miyake 2025).

C‐type domains have between 110 and 130 amino acids and a structure composed of two α‐helices and two antiparallel β‐sheets, containing between six and seven β‐strands. The calcium ions (between one and four per domain) are coordinated by amino acids present in a long loop between the β2‐ and β3‐strands, which is also responsible for carbohydrate recognition. The primary site of interaction with carbohydrates and other molecules is located in the upper region of the domain and contains a calcium ion. The other ions are related to secondary sites or the structural stability of the protein (Zelensky and Gready 2005).

In general, three motifs are responsible for the carbohydrate‐binding capacity of C‐type lectins. The WND (Trp‐Asn‐Asp) motif, through asparagine and aspartate residues, together with another amino acid with a carbonyl side chain, establishes coordination with Ca^2+^. The EPN (Glu‐Pro‐Asn) motif recognizes mannose, glucose, fucose, and N‐acetylglucosamine (GlcNAc) via the equatorial 3‐OH and 4‐OH groups, while QPD (Gln‐Pro‐Asp) recognizes galactose and N‐acetylgalactosamine (GalNAc) via the equatorial 3‐OH and axial 4‐OH groups. In both cases, the carbohydrate is stabilized by a network of hydrogen interactions with the carboxyl and amide groups of these motifs (Keller and Rademacher 2020; Zelensky and Gready 2005). The motifs contribute to the coordination of the ion, but also compose the CRD, allowing interactions with two adjacent monosaccharide hydroxyls (Huang et al. 2015; Zelensky and Gready 2005).

**FIGURE 1:**
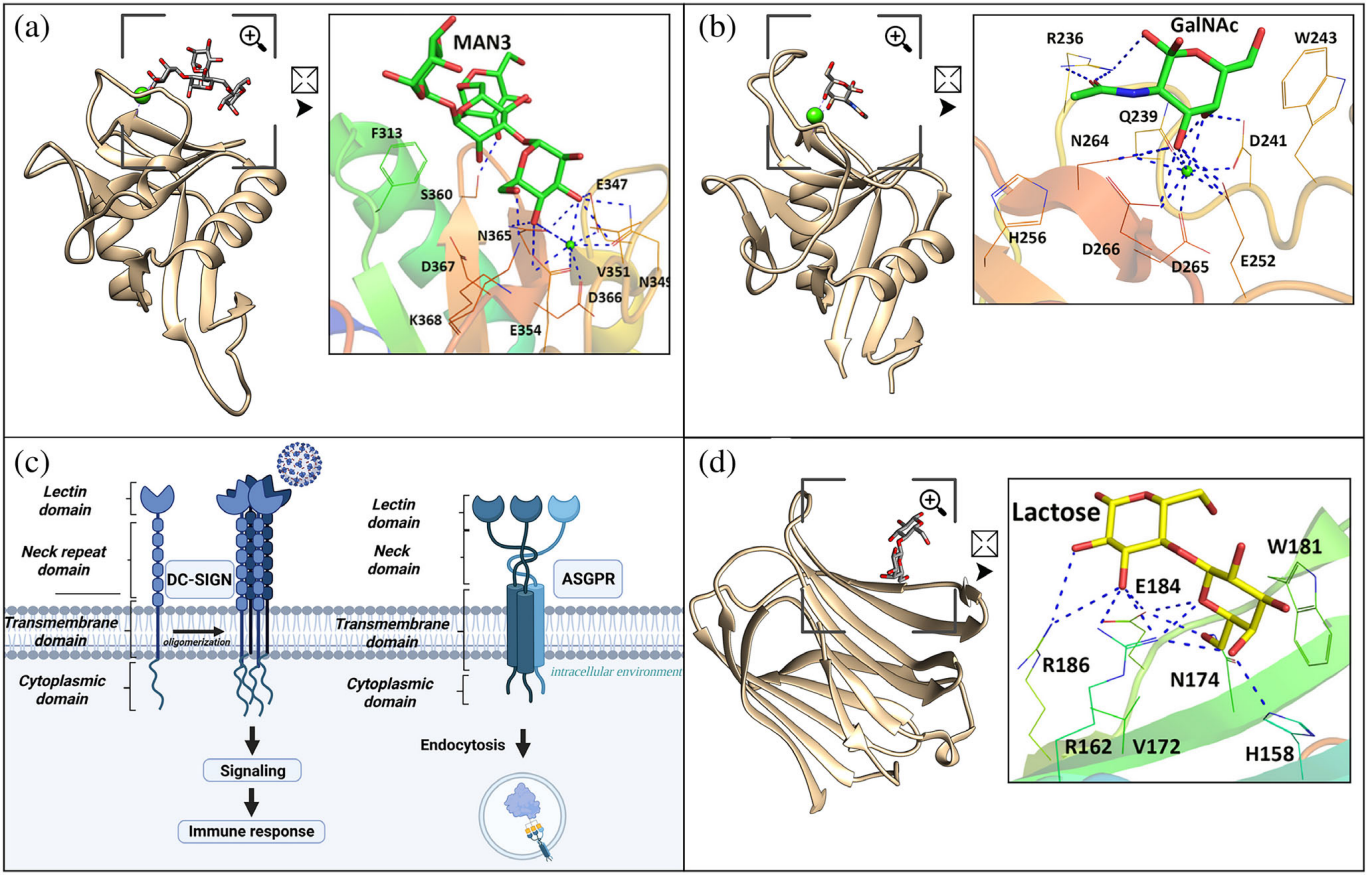
Structural characterization of DC‐SIGN, ASGPR, and Galectin‐3. (a) Carbohydrate recognition domain (CRD) of DC‐SIGN, a C‐type lectin, containing the E_347_P_348_N_349_ motif, complexed with Manα (1–3)[Manα (1–6)]Manα(1‐6)Man (Man4) (PDB ID: 1SL4); (b) CRD of ASGPR, also a C‐type lectin, containing the Q_239_P_240_D_241_ motif, complexed with N‐acetylgalactosamine (GalNAc) (PDB ID: 9G76); (c) schematic representation of the complete structural architecture of DC‐SIGN and ASGPR, showing the lectin domain (CRD), neck region, transmembrane domain, and cytosolic domain in the cell membrane; (d) CRD of Galectin‐3, a S‐type lectin containing the motif N_180_W_181_G_182_R_183_, complexed with lactose (PDB ID: 3AYE). In (a, b, d), the polypeptide chain is represented in cartoons. The amino acids involved in the interactions in the CRD and the carbohydrates are represented in stick format. In the cartoon representations, squares indicate regions of interest that have been magnified, as indicated by the symbol of a square with an arrow, for a detailed three‐dimensional analysis of the interaction.

Some C‐type lectins have structural domains other than the CRD that contribute to dimerization or oligomerization, essential for their biological functions. Stem and coiled‐coil domains promote the formation of functional multimers in groups, such as collectins and DC‐SIGN (Dendritic Cell‐Specific Intercellular Adhesion Molecule‐3‐Grabbing Non‐integrin) (Auriti et al. 2017; Dos Santos et al. 2017; Pederson et al. 2014). Collectins, such as MBL (mannose‐binding lectin), act as opsonins, promoting phagocytosis and activating the complement lectin pathway, which is important in innate immunity. MBL forms large oligomers composed of identical polypeptide chains (Fujita et al. 2004; Jensen et al. 2005). Three of these chains join together to form subunits, which then associate to form larger oligomers. Each subunit has a cysteine‐rich N‐terminal domain responsible for the formation of essential disulfide bonds, followed by a collagenous domain responsible for interactions with Mannan‐binding lectin‐associated serine proteases (MASP proteins), a serine protease, an α‐helix (neck) region, which functions in oligomerization, and three CRDs (Gingras et al. 2011; Gregory et al. 2004). DC‐SIGN expressed in dendritic cells recognizes pathogen glycans and participates in the internalization of antigens and modulation of the immune response (Gupta et al. 2023; Stewart et al. 2024). In addition to the CRD (Figure 1a), DC‐SIGN has a cytoplasmic tail located inside the cell; it participates in intracellular signaling after DC‐SIGN binds to molecular targets. It also has a transmembrane domain that contains a hydrophobic region that anchors the protein in the plasma membrane and a neck domain that contains 7.5 repeats of 23 amino acids and is essential for the formation of tetramers to increase the binding affinity of the protein. Both are C‐type lectins involved in mannose recognition (Feinberg et al. 2001; Mitchell et al. 2001; Tabarani et al. 2009).

An example is the ASGPR (Asialoglycoprotein Receptor) (Figure 1b), which is highly expressed on the surface of hepatocytes and acts in blood homeostasis to clear out specific glycoproteins. More specifically, as a lectin, ASGPR binds to glycoproteins after the removal of sialic acid, leaving exposed terminal galactose or GalNAc residues (Meier et al. 2000; Mishra et al. 2020; Ramírez‐Cortés and Ménová 2025). In addition to the CRD, this protein has a cytoplasmic domain, which is important for protein localization and cellular interactions, a transmembrane domain, which anchors ASGPR to the cell surface, and a neck domain that connects the transmembrane domain to the CRD (Hofmeister et al. 2025).

Another example is CLEC4F, a C‐type lectin expressed in Kupffer cells (resident liver macrophages) (Jiang et al. 2021). This protein participates in the recognition of galactosylated glycans on circulating glycoproteins, contributing to the clearance of antigens and glycosylated material in the hepatic environment (Yang et al. 2013). In addition to the CRD, it has a cytoplasmic signaling domain, which participates in intracellular signaling pathways, a transmembrane helix, a hydrophobic region that anchors CLEC4F to the cell membrane, and a heptad neck region that forms a coiled‐coil structure, essential for stabilizing the formation of trimers and fundamental for the function of CLEC4F (Ouyang et al. 2020).

Thus, since some C‐type lectins depend on cooperation with other structural domains to modulate their oligomerization, binding specificity and functional activity, they can be classified as chimerolectins (Figure 1c).

### 2.2 Galectin‐3, a chimeric S‐type lectin

Galectin‐3 is a 29‐ to 35‐kDa protein that is structurally unique among vertebrate galectins. It is composed of a single polypeptide chain that forms two distinct domains: an atypical N‐terminal domain (ND) and a CRD in the C‐terminal region (Dussouy et al. 2020; Pia Lenza et al. 2025).

ND is composed of 110–130 amino acids and contains homologous repeats rich in proline and glycine with a consensus sequence, Pro‐Gly‐Ala‐Tyr‐Pro‐Gly‐X‐X‐X (Newlaczyl and Yu 2011). The ND is essential for the biological activity of galectin‐3 as it partially participates in binding to oligosaccharides, together with the CRD (Xue et al. 2017). The ND is also responsible for the oligomerization and cellular localization of galectin‐3, which has a small, highly conserved sequence of 12 amino acids that include the Ser6 and Ser12 phosphorylation sites that regulate these two factors (Haudek et al. 2010; Lin et al. 2017; Zhao et al. 2024). The C‐terminal domain, the CRD (Figure 1d), is composed of approximately 130 amino acids organized in a β‐sandwich structure with two antiparallel β‐sheets responsible for the lectin activity of galectin‐3 (Seetharaman et al. 1998; Raics et al. 2022). Compared to the intact protein, the CRD, which is isolated from other domains, has a higher affinity for carbohydrates, suggesting that the ND may regulate access to the binding site and its activities (Zhao et al. 2021).

The CRD cavity is delimited by four structural loops that act in the orientation and interaction with carbohydrates (Saraboji et al. 2012). The loop, containing residue His158, participates in the formation of hydrogen bonds with the hydroxyl groups of galactose. Next to this, in the same loop, is Asn160, which is also essential in stabilizing the interaction via hydrogen bonds with the 3‐OH and 4‐OH groups of galactose. In a second loop, Arg162 forms multiple hydrogen bonds and electrostatic interactions with polar groups of the carbohydrate. This arginine promotes the correct alignment of the sugar molecule within the binding cavity. Asn174 can interact with non‐galactosidic portions of the ligand, such as the glucose residue present in lactose, reinforcing the stability of the interaction (Saraboji et al. 2012). A third loop contains the aromatic residue Trp181, responsible for performing hydrophobic stacking with the galactose ring, promoting, in turn, a CH–π type interaction, which confers greater specificity and affinity to recognition (Diehl et al. 2024). Also in this circuit is Glu184, which establishes hydrogen bonds with the 4‐OH group of galactose, crucial for distinguishing galactose from other monosaccharides. The combination of these interactions allows galectin‐3 to recognize with high affinity carbohydrates that contain galactose in the β configuration, such as lactose and other terminal glycans of glycoproteins and glycolipids (Diehl et al. 2024; Saraboji et al. 2012; Su et al. 2015). A highly conserved motif present in the CRD is NWGR (Asn214‐Trp215‐Gly216‐Arg217), similar to the BH1 domain of the Bcl‐2 family involved in the anti‐apoptotic activity and self‐association of galectin‐3 (Akahani et al. 1997; Zhang et al. 2025).

The binding specificity of galectin‐3 is primarily directed toward N‐acetyllactosamine (LacNAc), even while its structure allows for the accommodation of longer oligosaccharides, such as polylactosamines (Joeh et al. 2020; Kamili et al. 2016; Salomonsson et al. 2010). Through these domains, this versatile lectin contributes to several biological functions, such as immune regulation, proinflammatory response, apoptotic regulation, pathogen recognition, and cancer progression (Capone et al. 2021; Liu and Stowell 2023; Stojanovic et al. 2023; Wang et al. 2024). The presence of multiple domains allows galectin‐3 to participate in complex interactions and perform diverse functions in cell recognition and signaling. This lectin can interact with components of the extracellular matrix (ECM), such as laminin, fibronectin, and integrins, in interactions mediated by the CRD (Nangia‐Makker et al. 2022; Sedlář et al. 2021). Thus, galectin‐3 acts as an external “functional domain” that promotes protein activity. In addition, galectin‐3 interacts with Toll‐like receptors. Although it lacks a Toll/Interleukin‐1 receptor/Resistance protein (TIR) domain, it can modulate the signaling of TLRs, such as TLR4, during inflammatory processes, acting as an adaptor molecule (Burguillos et al. 2015; García‐Revilla et al. 2022; Liu et al. 2022; Zhou et al. 2018).

### 2.3 Enzymatic lectins

**FIGURE 2:**
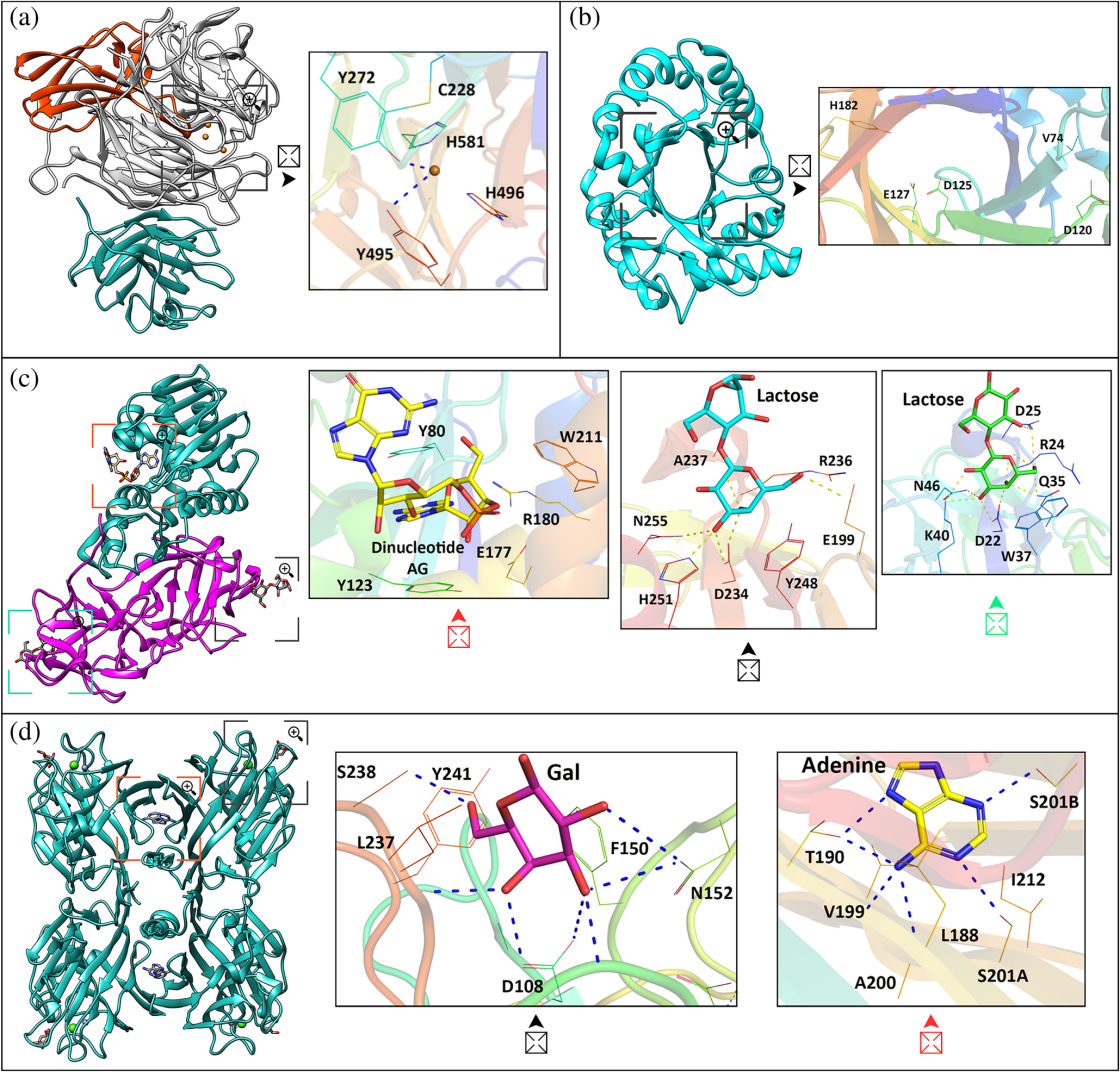
Structural characterization of enzymatic lectins: Galactose oxidase (GOase) from *Fusarium* sp., a chimerolectin from *Parkia platycephala* (PPL2), ricin from *Ricinus communis*, and a lectin II from *Dolichos lablab* (DLL‐II). (a) Structure of GOase, containing the lectin domain in green, the active site of the enzyme in gray, including the copper ion (brown sphere) that is essential for catalytic activity and is coordinated by four amino acids, and the Ig‐like domain in red (PDB ID: 2VZ3); (b) structure of PPL2, represented in cyan blue, containing a GH18 family domain, with (α/β)_8_ barrel architecture, highlighting the catalytic region (PDB ID: 2GSJ); (c) structure of ricin, composed of the RTA subunit, in green, with N‐glycosidase activity, complexed with an AG dinucleotide, and the RTB subunit, in pink, which has two lectin domains capable of interacting with lactose (PDB ID: 3RTJ); (d) structure of the DLL‐II tetramer, in green, complexed with galactose in the CRD and with adenine in the internal site of the tetramer (PDB ID: 3UJO). The polypeptide chains are represented in cartoon format. The amino acids involved in the interactions in the CRD and the carbohydrates are represented in stick format. In the cartoon representations, squares indicate regions of interest that have been magnified, as indicated by the symbol of a square with an arrow, for a detailed three‐dimensional analysis of the interaction. Squares of different colors were used to indicate different regions, and the enlarged figures are represented by symbols of the same color as the respective squares.

Galactose oxidase (GOase) from *Fusarium* spp. is a protein composed of 639 amino acids organized into three main domains involved in galactose metabolism (Figure 2a) (Cao et al. 2023; Firbank et al. 2001). In the N‐terminal region, the first domain is the carbohydrate‐binding module (CBM32, residues 1–155) responsible for the recognition of galactose in glycoconjugates. Although the structural basis of the interaction of CBM32 of GOase has not been fully elucidated, it generally involves such residues as Trp, Tyr, Asp, and Asn in its interactions. This module has a β‐sandwich structure with two overlapping antiparallel β‐sheets, one with five and the other with three β‐strands. The second domain (residues 156–552) in the central region forms a seven‐bladed β‐propeller and houses the active center of the enzyme, including the copper ion essential for catalytic activity, which by the oxidation of galactose to aldehyde produces hydrogen peroxide (Abbott et al. 2008; Nozaki et al. 2025). The copper ion is coordinated by four amino acids. Tyr272 and Tyr495 are involved in the formation of a tyrosyl radical essential for catalytic activity, and His496 and His581 contribute to the coordination of the copper ion at the active site. In addition, the Cys228 residue forms a thioether bond with Tyr272, stabilizing the tyrosyl radical necessary for the oxidation of D‐galactose (Borman et al. 1997; Mathieu et al. 2022). The third domain in the C‐terminal region (residues 553–639) consists of an Ig‐like β‐sandwich fold that comprises two β‐pleated sheets, one with three and the other with four antiparallel β‐strands, and acts as a “lid” over the catalytic domain, contributing to the stability of the global structure of the enzyme and the coordination of copper from residue His581 (Keser et al. 2025).

A second type of enzyme, lectin, has a chitinase domain that breaks the β‐1,4 bonds between the GlcNAc units in chitin, thus acting in the defense of plants against fungi and other pathogens. PPL2, a chimerolectin from *Parkia platycephala*, a legume species, has a domain belonging to the GH18 family of glycoside hydrolases (Figure 2b). It presents an (αβ)8 barrel with conserved β‐strands and a critical hydrogen bond network among residues Asp120, Gly121, and Val74, which helps to stabilize the active site. The presence of the b3 and b4 motifs in the PPL2 structure is characteristic of the GH18 family and is essential for its enzymatic function. The most notable structural divergence of PPL2 occurs in the active site loops, which connect neighboring β‐strands. With the exception of the β6‐α6 loop, the active groove loops of PPL2 are highly conserved. These variations may explain the interaction of this site with oligosaccharides containing N‐acetylglucosamine, which influences its lectin activity, including erythrocyte agglutination, as well as its enzymatic activity. The catalytic domain consists of residues Glu127 (proton donor for glycosidic bond), Asp125, and Tyr182 (stabilizing intermediates) located in the β4‐α4 and β6‐α6 loops (Cavada et al. 2006). These residues are highly conserved in GH18 chitinases and participate in catalysis. Other similar lectins include the ConB lectin from *Canavalia ensiformis*, which has a structure and sequence similar to that of GH18 chitinase but no enzymatic activity (Hennig et al. 1995) and the lectin TCLL from *Tamarindus indica* (Patil et al. 2013). In addition, some plant proteins are similar to chitinases GH18 and GH19 with a catalytic domain and covalently anchored chitin‐binding hevein domains, such as that observed in the proteins of *Hevea brasiliensis* and *Simarouba glauca* (Balu et al. 2020; Martínez‐Caballero et al. 2014).

The third group is comprised of lectins with N‐glycosidase activity, also known as RIPs (Ribosome Inactivating Proteins) type 2. In addition to binding to carbohydrates and agglutinating erythrocytes, RIPs are considered potent toxins that, as the name suggests, inactivate ribosomes, thus performing a general defensive role for plants (Dougherty and Hudak 2022). The best known are Ricin (*Ricinus communis*) and Abrin (*Abrus precatorius*). Ricin consists of two polypeptide chains (Figure 2c). The first is RTA (residues 1–267, ~32 kDa), a catalytic subunit with N‐glycosidase activity responsible for irreversibly inactivating a specific adenine (A4324) of 28S ribosomal RNA. The second is RTB (residues 268–576, ~34 kDa), a lectin domain capable of binding galactose or N‐acetylgalactosamine linked by a disulfide bridge (Cys259 of RTA and Cys4 of RTB). RTA has a typical RIP fold dominated by α‐helices with antiparallel β‐sheet elements. The active site is formed by residues Tyr80 and Tyr123 responsible for CH–π stacking with the adenine removed from the rRNA; Glu177, which acts as an acid–base in catalysis and facilitates cleavage of the adenine from rRNA; Arg180 stabilizes the negative charges of the rRNA, and Trp211 participates in the recognition of specific adenine (Goto et al. 2023; Rutenber et al. 1991). The RTB is composed of repeated globular domains, each with a carbohydrate recognition site. Each domain is formed by three antiparallel β‐sheets, each with four β‐strands (β‐prism type II) organized around a pseudo‐triangular axis. Domain I (N‐terminal of RTB) is composed of residues Asp22, Asp25, Gln35, Trp37, Lys40, and Asn46, among which Trp37 and Asp25 are critical for CH–π stacking interactions and sugar recognition, while the others contribute to hydrogen bonding, electrostatics, and van der Waals interactions. Domain 2 (C‐terminal) is composed of residues Glu199, Asp234, Arg236, Ala237, Tyr248, His251, and Asn255, among which Tyr248 and His251 directly participate in the recognition of galactose through CH–π interactions and hydrogen bonds. Despite their similarity, domain 2 has a higher affinity for galactosides than domain 1 (Lord et al. 1994; Polito et al. 2019). Abrin is a homologous protein with conserved domains in relation to Ricin, but its differences in amino acid composition ensure greater affinity for human glycoconjugates and greater catalytic efficiency, making Abrin more toxic (Bagaria and Karande 2014; Tahirov et al. 1995).

*Dolichos lablab* lectin II (DLL‐II) has two types of chains, α (281 residues) and β (263 residues), which compose its structure. Each DLL‐II subunit exhibits the typical fold of legume lectins, a β‐sandwich with a flat β‐sheet containing six strands connected by loops to a second curved β‐sheet with seven strands (Figure 2d). It presents a metal‐binding site with Ca^2+^ and Mn^2+^ ions coordinated by amino acids Glu146, Asp148, Phe150, Asn152, Asp156, and His161, in addition to four water molecules. The α chain is identical to the β chain, except for containing an additional C‐terminal helix located within the tetramer formed by the association of these chains. The tetramer has a type II (or canonical) interface formed by the lateral association of two equivalent flat β‐sheets, creating a continuous β‐sheet with 12 strands. Additionally, it has a modified X1‐type interface; the presence of an α‐helix makes it a DB58‐type interface. The interaction domain with β‐galactose (β‐Gal) is formed by the following residues: Asp108, which forms hydrogen interactions with the O3 and O4 atoms; Gly126, Asn152, and Leu237, which form hydrogen bonds with the O3 atom; Ser238, which forms hydrogen bonds with the O6 atom; Phe150, which contributes to the hydrophobic interaction with the β face of galactose; and Leu237 and Ser238, which participate in van der Waals interactions. At the interface between the α and β chains, a hydrophobic pocket is formed that facilitates interactions with adenine molecules. The interaction of adenine with DLL‐II is mediated by hydrogen bonds involving residues Ser201 and Ile212, which interact with atoms N‐1 and N‐3; Thr190, Val199, and Ala200, which contribute to the interaction with atom N‐6; and Leu188, which interacts with atoms N‐6 and N‐7 (Shetty et al. 2013). DLL‐II also exhibits polyphenol oxidase (PPO) activity, but its structure and geometry are quite different from that of canonical PPOs, which have a metallic center consisting of two copper nuclei (Rao et al. 2012). In DLL‐II, data indicate that only one copper (or manganese) ion is required for PPO activity, whereas Ca^2+^ is required for lectin activity. This difference suggests that DLL‐II may operate by an alternative mechanism, which is yet to be confirmed experimentally (Yashavanth et al. 2018). This lectin may perform carbohydrate recognition and binding for signaling and defense, as well as catalysis related to oxidative defense.

Although it is not yet well explored, some lectins have conjugated TIR domains (Toll/Interleukin‐1 Receptor Homology Domain) (Santamaría et al. 2019). This domain has a globular shape comprising a five‐stranded parallel β‐sheet surrounded by four α‐helical structures. In contrast to animal TIR domains, plants, such as Arabidopsis, have an additional helix (αD3), which is important for oligomerization and domain function, such as that observed in the RPS4 protein (Bernoux et al. 2011; Chan et al. 2010). A predicted chimerolectin from *Arabidopsis thaliana*, PP2‐A5, is a protein that contains a TIR domain at the N‐terminal region and a PP2 (Phloem Protein 2) domain at the C‐terminal region. The typical PP2 domain presents a β‐sandwich fold composed of two antiparallel sheets that stack to form the globular structure. The catalytic function of the TIR domain and the recognition of carbohydrates by the PP2 domain have not yet been characterized. The PP2‐A5 gene is induced in response to attack by the generalist phytophagous mite *Tetranychus urticae*, and its expression is related to the perception and response to biotic stresses (Santamaría et al. 2019). The combination of these two domains in a single protein may represent a mechanism to control gene expression during the immune response since the expression of this chimerolectin is associated with the fine regulation of hormonal and transcriptional responses in plants under attack by mites.

### 2.4 Siglecs

**FIGURE 3:**
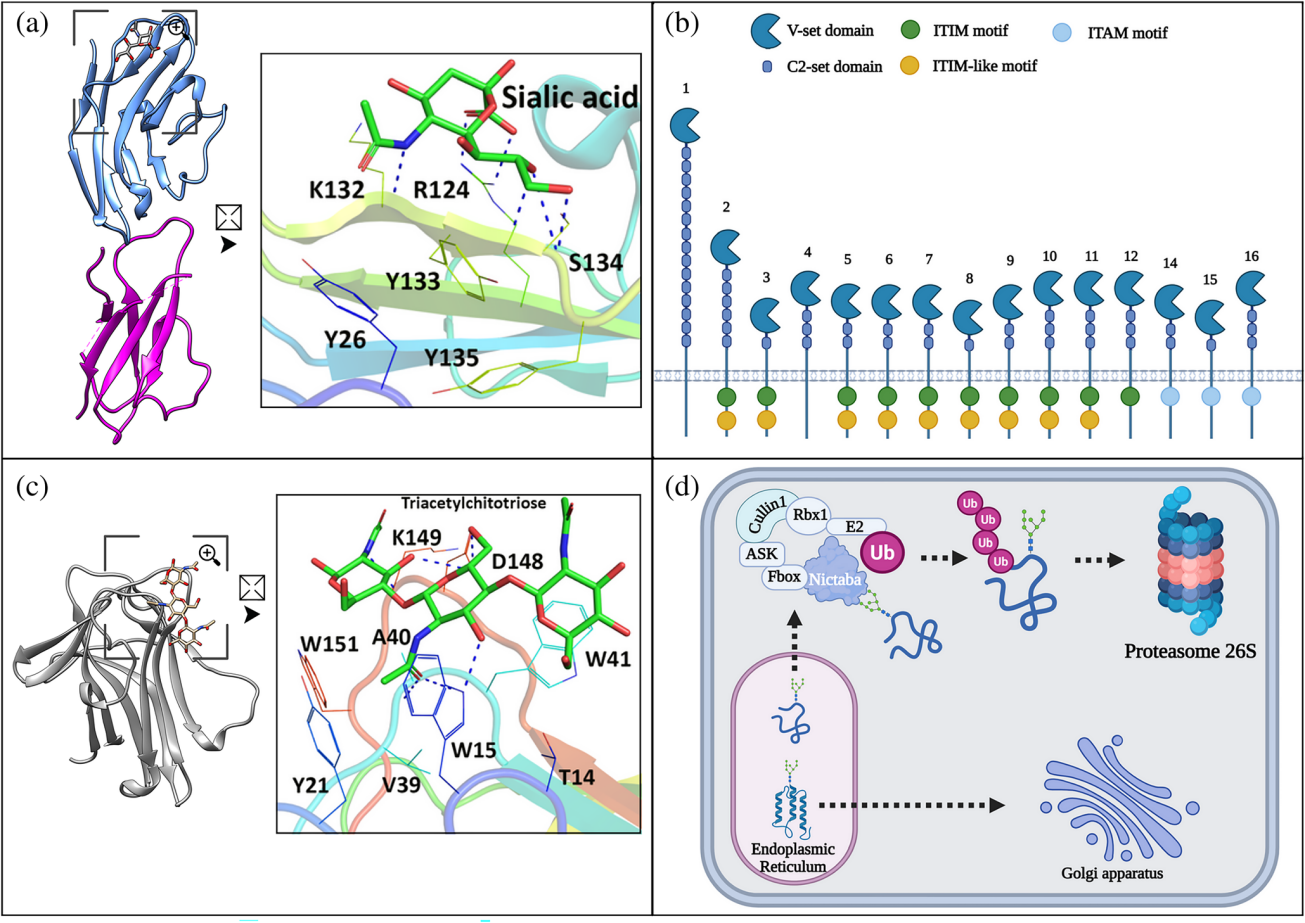
Structural and functional characterization of Siglecs and lectins with Nictaba‐Fbox domains. (a) Structure of Siglec‐5, with the V‐set domain in blue complexed with a sialic acid derivative and the C2‐set domain in magenta, both with Ig‐like conformation (PDB ID: 2ZG1); (b) schematic representation of the structures of different Siglecs, highlighting the presence of the V‐set domain, the number of repeats of the C2‐set domains in each type and the presence of the ITIM, ITIM‐like and ITAM signaling motifs; (c) structure of the Nictaba domain, in gray, complexed with triacetylchitotriose (PDB ID: 8AD2); (d) schematic representing the hypothetical mechanism of action of F‐box‐Nictaba proteins. Correctly folded proteins are transported from the endoplasmic reticulum (ER) to the Golgi complex. On the other hand, misfolded proteins that present the N‐glycan Man_3–9_GlcNAc_2_ are translocated to the cytosol. In the cytosol, the Nictaba‐Fbox protein, a member of the SCF complex, specifically recognizes this glycan and promotes the labeling of the protein with ubiquitin. The ubiquitinated protein is then targeted for degradation by the 26S proteasome. In (a, c), the polypeptide chains are represented in cartoon format. The amino acids involved in the interactions in the CRD and the carbohydrates are represented in stick format. In the cartoon representations, squares indicate regions of interest that have been magnified, as indicated by the symbol of a square with an arrow, for a detailed three‐dimensional analysis of the interaction.

Siglecs (sialic acid‐binding immunoglobulin‐like lectins) are chimerolectins that act as cell surface receptors and play an important role in modulating immune responses (Movsisyan and Macauley 2020). Structurally, they have an extracellular portion, composed of V and C2 domains, a transmembrane region, and a cytoplasmic tail (Figure 3a). The extracellular portion is composed of a variable number of Ig‐like C2‐set domains. The number of these domains, typically ranging from 1 to 16, mainly influences the spacing and orientation of the binding domain in relation to the cell membrane, thereby modulating the mode of interaction of Siglecs with their molecular targets (Figure 3b). The rising number of domains correlates with the rising probability of Siglec interacting in trans mode; that is, with glycans present in another cell. Siglec‐1 (sialoadhesin), for example, has 15 C2‐set domains and tends to interact with glycans in *trans* mode. Siglecs with fewer extracellular domains, such as Siglec‐3, ‐8, and ‐15, generally bind to glycans present in the cell itself (*cis* mode) (Angata et al. 2004; Crocker et al. 2007; Jame‐Chenarboo et al. 2024; Klaas et al. 2023).

Furthermore, these lectins possess an N‐terminal V‐set‐type domain, which is structurally related to the variable domain of antibodies, and it is responsible for the recognition of sialic acid in glycoconjugates (Prenzler et al. 2023). Sialic acid binding strongly depends on specific interactions mediated by conserved residues in the V‐set domain. In the case of Siglec‐1 (Siglec‐1), Arg116 forms a salt bridge with the carboxyl group of sialic acid, while the acetyl group of the carbohydrate interacts with the indole ring of Trp21. Additionally, the side chain of Trp125 interacts with the glycerol moiety of sialic acid. Similarly, Siglec‐5 also binds to sialic acid through a salt bridge involving Arg124 and the carboxyl group of sialic acid. Other important interactions include hydrogen bonds between Lys132 and Ser134 and the functional groups of sialic acid, as well as van der Waals interactions between the aromatic residue Tyr133 and the C9 position of the carbohydrate. As suggested by their interactions, the structures of these binding sites, although conserved, present variations that can modulate the affinity and specificity of each Siglec for different sialylated glycans (Hartnell et al. 2001; May et al. 1998). Structurally, the C2‐set and V‐set domains share the Ig‐like fold formed by a β‐sandwich consisting of two antiparallel β‐sheets stabilized by hydrogen bonds, often with a disulfide bridge connecting conserved regions. This β‐sandwich fold is predominantly composed of nine β‐strands organized into two planes that pair with each other, forming a robust and highly conserved structure. The transmembrane region of Siglecs is composed of a single hydrophobic α‐helix segment, which anchors the protein in the plasma membrane (Duan and Paulson 2020; Lenza et al. 2023).

Siglecs may also possess intracellular domains responsible for signaling that are essential for activating or inhibiting immunomodulatory responses. The ITIM (immunoreceptor tyrosine‐based inhibitory motif) motif with the consensus sequence (I/V/L/S)‐X‐Y‐X‐X‐(L/V) is located in the cytoplasmic tail of several Siglecs. In this sequence, I/V/L/S represent hydrophobic amino acids, while Y is a tyrosine critical for phosphorylation by Src family kinases after Siglec binds to its target, and X can be any amino acid. The phosphorylated tyrosine serves as a recruitment platform for SHP family phosphatases, such as SHP‐1 and SHP‐2, through their SH2 domains. These phosphatases act by removing phosphate groups from signaling enzymes associated with receptors that activate immune response, interrupting the cellular activation cascade (Bornhöfft et al. 2018; Crocker et al. 2007; Pillai et al. 2012; Wu et al. 2022).

In addition to ITIM, similar motifs may increase phosphatase recruitment and modulate signaling. Most Siglecs, such as Siglec‐2, ‐3, ‐5, ‐7, ‐9, ‐10, and ‐11, contain ITIM motifs. On the other hand, Siglec‐14, ‐15, and ‐16 have ITAM (immunoreceptor tyrosine‐based activation motif)‐activating motifs not directly present in Siglec; instead, they are present in associated adaptor proteins, such as DAP12 and DAP10 (Gianchecchi et al. 2021; Kukan et al. 2024; Suematsu et al. 2019). The ITAM motif is composed of the Y‐X‐X‐L/I‐X_6–8_‐Y‐X‐X‐L/I sequence in which Y residues are essential tyrosines for phosphorylation, L/I are hydrophobic residues, and X_6–8_ represents a spacing of 6 to 8 residues. Upon activation of Siglec, the two tyrosines of ITAM are phosphorylated by Src family kinases, creating docking sites for kinases with SH2 domains, which, in turn, phosphorylate other proteins of the signaling cascade, promoting the activation of the immune response. Activated Siglecs also have a positively charged residue, such as arginine in the transmembrane region, facilitating interaction with adaptive proteins that contain ITAM (Crocker et al. 2007; Kukan et al. 2024).

The architecture of Siglecs allows these chimerolectins to modulate immune responses, either by inhibition or activation, depending on the cellular context and the type of ligand present. Siglecs without ITIM or ITAM motifs, such as Siglec‐1, play a role in homeostasis and immune regulation, acting as pattern recognition receptors that compete with other Siglecs or receptors that bind sialic acid, passive modulators of signaling, or facilitators of cell–cell interactions.

### 2.5 F‐box lectins with Nictaba‐like domain

Chimerolectins containing an F‐box domain associated with a Nictaba‐like domain regulate responses to biotic and abiotic stresses in plants through the recognition of glycosylations and cell signaling (Figure 3c) (Stefanowicz et al. 2012; Wen et al. 2023). The F‐box domain, which is structurally composed of approximately 50 amino acids, mainly functions as a mediator of protein–protein interactions (PPIs). It adopts a conformation composed of three α‐helices organized in a right‐handed supercoiled helical arrangement, favoring interaction with the Skp1 protein, a component of the SCF (Skp1–Cullin–F‐box) ubiquitin ligase complex responsible for the selective degradation of proteins via the proteasome (Figure 3d). This domain is typically located in the N‐terminal region of this class of chimerolectins (Eggermont et al. 2017; Kipreos and Pagano 2000; Schulman et al. 2000; Stefanowicz et al. 2015).

The Nictaba‐like domain is located in the C‐terminal region and adopts a β‐sandwich structural fold, totaling approximately 12 β‐strands. A distinctive element is the presence of an accessory hairpin β‐loop, which contributes to the stability and topology of the domain. Based on the Nictaba structure, the carbohydrate‐binding site is located in a cavity on the domain surface and specifically recognizes N‐acetylglucosamine (GlcNAc) residues. Two main‐chain nitrogens of Ala40 and Trp41 form hydrogen bonds with the carbonyl oxygen of the acetyl group. The O3 atom of GlcNAc establishes a hydrogen bond with the side‐chain nitrogen of Trp15. A fourth hydrogen bond occurs between the carbohydrate nitrogen and the main‐chain oxygen of Asp148. In addition, CH–π stacking interactions occur within the indole ring of Trp41. Other residues that form the binding pocket include Thr14, Tyr21, Val39, Lys149, and Trp151 (Bloch et al. 2024).

To date, no experimental structure of an F‐box domain‐containing lectin has been solved, leaving only modeled structures available. This represents a challenge owing to the low expression of these proteins and their rapid degradation.

### 2.6 Pathogen lectins

**FIGURE 4:**
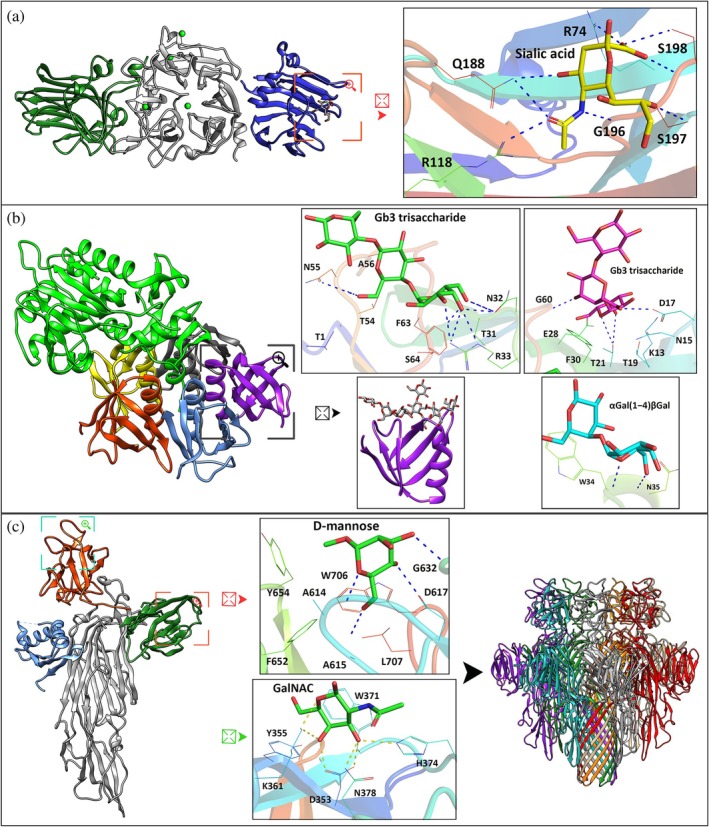
Structural characterization of pathogen chimerolectins. (a) Structure of the *Vibrio cholerae* sialidase‐lectin (VCNA), with the central catalytic domain represented in gray, containing a calcium ion (green sphere), and two flanking lectin domains, one in green and the other in blue. The CRD located in the N‐terminal region (in blue) is complexed with sialic acid (PDB id: 1W0P); (b) structure of the Shiga toxin from *Shigella dysenteriae*, composed of an A subunit, with N‐glycosidase activity (in green), and a homopentamer corresponding to the B subunit (represented in yellow, gray, purple, blue and red), containing three CRDs per monomer, with specificity for globotriaosylceramide derivatives (PDB ids: 1R4Q, 1BOS); (c) structure of the *V. cholerae* cytolysin (VCC) monomer, containing the prodomain (present only in the precursor) in blue, the cytolysin domain in gray, a β‐trefoil lectin domain in red, and a β‐prism lectin domain in green (PDB ID: 1XEZ). The β‐trefoil domain is shown complexed with N‐acetylgalactosamine (GalNAc) (PDB ID: 4OWK) and the β‐prism domain with mannose (PDB ID: 4GX7). The quaternary structure of VCC, corresponding to its heptamer, is also shown (PDB ID: 3O44). The polypeptide chains are represented in cartoon format. The amino acids involved in the interactions in the CRD and the carbohydrates are represented in stick format. In the cartoon representations, squares indicate regions of interest that have been magnified, as indicated by the symbol of a square with an arrow, for a detailed three‐dimensional analysis of the interaction.

Sialidase‐lectin proteins are multifunctional proteins that combine two main functional domains: the sialidase (or neuraminidase) domain, which is responsible for the cleavage of sialic acid (N‐acetylneuraminic) residues from glycoconjugates, and the carbohydrate‐binding domain with affinity for glycans containing sialic acid or related carbohydrates (Engibarov et al. 2025). The best‐known representatives of this group are present in pathogenic bacteria, such as *Vibrio cholerae* (VCNA), and they are associated with bacterial virulence and the modulation of epithelial barriers (Kaisar et al. 2021; Moustafa et al. 2004). VCNA has two carbohydrate‐binding domains, one in the N‐terminal region and the other in the C‐terminal region, which flank the catalytic domain (Figure 4a). The 83 kDa protein has a central catalytic domain with a β‐propeller fold with six β‐strands, characteristic of neuraminidases, and two flanking lectin domains, both with a β‐sandwich topology composed of an arrangement of antiparallel β‐sheets, one with seven and the other with six β‐strands (Crennell et al. 1994; Moustafa et al. 2004). The first domain, which is located at the N‐terminal region (residues 25–216), contributes the outer strand of the sixth β‐sheet of the catalytic domain. The first two β‐sheets of the catalytic domain are then completed before the chain projects again to form the second lectin domain (residues 347–543). After this second domain, the chain returns to complete the remaining four β‐sheets of the catalytic domain, ending the structure with a small α‐helix at the C‐terminus. The interaction of VCNA with the inhibitor 2‐deoxy‐2,3‐didehydro‐N‐acetylneuraminic acid (Neu5Ac2en) demonstrated that the catalytic site is composed of a triad of arginines (Arg224, Arg635, and Arg712), responsible for the interaction with the carboxylate group of Neu5Ac2en. Tyr740 is positioned close to the C‐2 carbon of Neu5Ac2en and, together with Glu619, participates in the activation of the nucleophilic water molecule or stabilization of the transition state. Ile225 establishes a hydrophobic interaction with the C‐2 carbon of the ligand, contributing to the proper positioning of the substrate in the catalytic site. Neu5Ac2en is accommodated in a cavity delimited by Trp311, Gln317, and Pro251, and by Asp292 and Asn545. The domain also presents a Ca^2+^ ion located on the surface coordinated by residues Asp245, Asp250, Trp311, and Asn318, maintaining three united loops essential for its activity (Crennell et al. 1994; Moustafa et al. 2004). The lectin domain (N‐terminal) interacts with sialic acid through Arg74 and Arg118, which contribute to the electrostatic stabilization of the carboxylate group. Gln188 establishes hydrogen bonds with hydroxyls (C4, C7, or C8), and Gly196 establishes hydrogen bonds with nitrogen atoms, while Ser197 and Ser198 establish hydrogen bonds with hydroxyls of the lateral tail (C7, C8, and/or C9) (Moustafa et al. 2004). Other lectin sialidases with a similar configuration can be found in *Streptococcus pneumoniae* (NanA, NanB, and NanC) (Gut et al. 2008; Sharapova et al. 2021); in *Clostridium perfringens*, whose protein has a lectin domain that presents differences in interactions with sialic acid, suggesting the existence of subfamilies within the CBM40 family (Ribeiro et al. 2016); and in *Micromonospora viridifaciens*, whose protein contains an immunoglobulin‐like (Ig‐like) domain and a galactose‐binding domain in addition to the GH33 catalytic domain (Gaskell et al. 1995). The *trans*‐sialidases of *Trypanosoma* spp. have a GH33 catalytic domain and a carbohydrate‐binding domain structurally similar to that of the CBM40 family, thus allowing the transfer of sialic acid residues between glycoconjugates (Buschiazzo et al. 2002; da Costa et al. 2021; Waespy et al. 2022).

Shiga toxin (Stx) is responsible for inhibiting protein synthesis through the catalytic inactivation of 60S ribosomal subunits, as identified in *Shigella dysenteriae* (Lee et al. 2020). Its basic structure is composed of a catalytic subunit A and a pentamer of subunit B responsible for cell recognition. Its subunit B binds to the Gb3 receptor in cells, facilitating the entry of subunit A, which results in inactivating the ribosome by removing an adenine from the 28S RNA (Figure 4b) (Kulczyk et al. 2023; Ling et al. 1998). This activity causes cell death, inflammation, and vascular damage (Jandhyala et al. 2012). The A subunit has two parts that can be separated by proteolytic cleavage. A1 contains the active site responsible for the N‐glycosidase activity of the toxin, containing the glutamic acid amino acid at position 167, which acts as the catalytic mechanism (Shiga‐like toxin I [SLT‐I]) (Hovde et al. 1988). A2 consists of a small α‐helical peptide that connects the A1 subunit to the B pentamer. A2 spans the central channel of the B pentamer, contributing to overall stability. A1 and A2 are joined by a disulfide bridge (Melton‐Celsa et al. 2002). The A1 region presents a typical structure of N‐glycosidase enzymes with a core formed by a mixed β‐sheet made up of six parallel and antiparallel strands and α‐helices distributed around it that form a globular domain housing the active site. This β‐sheet positions Tyr77 within the active site. The subunit also contains a double‐turn helix that houses Tyr114, important for the structure of the active site. Another relevant structural element is the helix‐turn‐helix motif, which houses Glu167, Ala168, and Arg170 at the end of the first helix where Glu167 functions as the catalytic residue essential for N‐glycosidase activity. The structure also includes a helix with corners. Each monomer of the Shiga toxin B subunit presents a fold composed of an internal α‐helix and six β‐strands. The sheets interact with each other to form the pentamer. The α‐helices are positioned on the inner face of the ring and form a central cavity that accommodates the α‐helix of A2 (Kulczyk et al. 2023; Melton‐Celsa 2014). The Shiga toxin B subunit pentamer has a total of 15 binding sites to the terminal trisaccharide of the Gb3 receptor (Gal1α1 → 4Gal2β1 → 4Glc; globotriaosylceramide (Gb3) with three sites per monomer). Therefore, the B subunit promotes multivalent binding, which dramatically increases the affinity for the target cell membrane. In the first site, hydrogen bonds involving Asp17, Thr21, Glu28, and Gly60 interact with atoms O4, O5, and O6 of Gal1 and atoms O3 and O6 of Gal2, as well as hydrophobic interactions with Leu29 and Phe30. In the second site, hydrogen bonds involving Asp16, Asn32, Arg33, Asn55, and Phe63 interact with atoms O2‐6 of Gal1 and O6 of Gal2, which is complemented by hydrophobic interactions with Thr1, Phe30, Thr31, Thr54, Asn55, Ala56, Gly62, and Ser64. In the third site, hydrogen bonds involving Trp34 and Asn35 interact with atoms O4‐6 of Gal1, while Trp34 also contributes important hydrophobic interactions (Ling et al. 1998). Other toxins share structural similarities with Shiga toxin, but with different mechanisms of action and molecular targets, such as in *E. coli* (Shiga toxin‐producing *E. coli* [STEC]) and cholera toxin (*Vibrio cholerae*) (Fraser et al. 2004; Sui et al. 2023; Vanden Broeck et al. 2007).

*Vibrio cholerae* cytolysin (VCC), an 80 kDa protoxin, can also be considered a chimerolectin. It assembles into a heptameric pore in target cell membranes after proteolytic cleavage and interaction with cell surface receptors via carbohydrate interaction (De and Olson 2011; Olson and Gouaux 2003). VCC is a modular protein organized into 3 distinct domains, each with a fold and an arrangement of secondary elements that confer specific functions to the toxin (Figure 4c) (Kathuria and Chattopadhyay 2018). At the beginning of the chain, a prodomain of ~15 kDa presents a fold characterized by a four‐stranded β‐sheet paired with three small α‐helices. It is present in an inactive VCC precursor and then cleaved by proteases for activation of the toxin. Following the prodomain is the cytolysin domain which constitutes the structural and functional core of the protein. With approximately 325 residues, this domain exhibits a fold that resembles that of α‐hemolysin. Its structure is predominantly formed by β‐sheets that are organized into two subdomains: a β‐sandwich where two antiparallel β‐sheets (one with four and the other with five strands) form a stable, stacked structure and a rim domain that presents extensive loops where aromatic residues and specific interactions are crucial for the domain's function (De and Olson 2011). Within this context, the pre‐stem assumes a central role in pore formation; in VCC, it is organized as an antiparallel β‐sheet composed of two strands that interact with the β‐sandwich subdomain (Kathuria and Chattopadhyay 2018). After the cytolysin domain, the protein incorporates two domains with lectin folds that facilitate interaction with carbohydrates. The first of these is the β‐trefoil domain. Each subdomain contains conserved motifs, especially Q × W features that refer to the B domain of ricin. This fold is composed of a combination of radially arranged β‐sheets, creating a clover‐shaped structure, and its interface with the cytolysin domain is reduced, suggesting a more flexible binding. The last domain, the β‐prism, is characterized by having three groups of antiparallel β‐sheets, each formed by four strands. This conformation is similar to that found in such lectins as jacalin. Connected to the β‐trefoil domain, the β‐prism domain presents a superficial pocket in which the binding of a molecule of β‐octyl glucoside and D‐mannose were observed (Olson and Gouaux 2003; Olson and Gouaux 2005). Residues such as Asp617, Tyr654, and Trp706 are arranged in a way that facilitates hydrogen interactions and aromatic stacking with carbohydrates, suggesting a role in anchoring the toxin to the cell surface (Rai et al. 2013). This multifaceted organization is essential for the VCC activation mechanism whereby proteolysis of the prodomain and reorganization of the domains culminate in the insertion of the pre‐stem into the membrane and the consequent formation of pores that lead to cell lysis (Kathuria and Chattopadhyay 2018). Cytolysin plays a central role in inducing inflammatory responses and cell death during infection. It activates a novel combination of Toll‐like receptors (TLR1/4) in addition to engaging TLR2, promoting proinflammatory signals in dendritic cells, macrophages and neutrophils (Gandhi et al. 2025).

### 2.7 Lectin receptor‐like kinases

Lectin receptor‐like kinases (LecRLKs) are important classes of transmembrane receptors in plants, acting as molecular sensors that recognize extracellular signals and activate specific cellular responses. These proteins play central roles in plant innate immunity, especially in the recognition of pathogen‐associated molecular patterns (PAMPs), such as fungal cell wall fragments. They also participate in adaptation to abiotic stresses, including salinity, drought, and extreme temperatures. In addition, they are involved in root development and growth, recognition of extracellular carbohydrates or glycoconjugates, and mediating plant‐microorganism interactions, both pathogenic and beneficial, such as symbiosis with mycorrhizae and rhizobia (Haider et al. 2021; Liu et al. 2018; Sun et al. 2020).

LecRLKs are membrane proteins composed of three main domains: an extracellular lectin‐like domain, a transmembrane domain, and a cytoplasmic domain with kinase activity. They are classified based on the lectin domain present, which can be L‐type, G‐type, C‐type, and LysM (Bellande et al. 2017; Buendia et al. 2018; Vaid et al. 2013). L‐type LecRLKs exhibit a membrane‐anchored β‐sandwich‐fold legume‐like domain and possess a conserved hydrophobic cavity that allows binding to complex glycans, plant hormones, and PAMPs (Osterne et al. 2024; Wang and Bouwmeester 2017). The G‐type domains contain a Galanthus nivalis lectin‐like (GNA‐like) domain with specificity for α‐D‐mannose in addition to accessory domains, such as SLG (S‐locus glycoprotein) associated with self‐incompatibility, PAN (Plasminogen/Apple/Nematode) involved in protein–protein and protein‐carbohydrate interactions, and EGF (Epidermal Growth Factor) potentially involved in the formation of disulfide bonds. All these domains are present in a β‐barrel fold structure with 12 β‐strands (Naithani et al. 2021; Van Damme 2022). The C‐type, which contains a calcium‐dependent lectin domain, is described in this review in section 2.1. Finally, some LecRLKs present a LysM domain that contains between 44 and 65 amino acids with conserved serine, threonine and aspartic acid residues, generally preceded by a signal region consisting of two α‐helices connected by a pair of antiparallel β‐strands, commonly known as a β‐α‐α‐β fold, but also called “β‐grasp” or “eubacterial lysin fold.” This fold forms a compact and hydrophobic, but flexible, core capable of accommodating different oligosaccharides. The LysM domain has specificity for N‐acetyl‐D‐glucosamine (GlcNAc), and it is capable of interacting with its derivatives, such as chitin and peptidoglycans (Abedi et al. 2021; Buist et al. 2008; Petutschnig et al. 2010; Yao et al. 2023).

The transmembrane domain is essential for localization to the plasma membrane and may be involved in ligand recognition and signal transduction. It consists of one or more hydrophobic α‐helices with about 20–25 amino acids that span the lipid bilayer of the plasma membrane. LecRLKs may have one to three TM domains; studies indicate different possible orientations of the extracellular and kinase domains, suggesting functional variations (Bi et al. 2016; Hohmann et al. 2017).

The cytoplasmic kinase domain generally has Ser/Thr kinase activity with conserved motifs, such as DIKPAN and GT(F/I/L)GYIAPE (Hervé et al. 1999) composed of two globular lobes: a smaller N‐terminal lobe dominated by β‐sheets (usually five antiparallel) and a larger C‐terminal lobe predominantly α‐helical. Evidence suggests that some members may also exhibit tyrosine kinase activity (dual activity) (Taylor et al. 2016). Between these lobes is the catalytic cleft where recognition and transfer of the phosphate group from ATP to the substrate occur. In addition to the β‐sheets and α‐helices, the secondary structure of the kinase domain includes highly conserved motifs. Enzymatic activity is favored by divalent cations (e.g., Mg^2+^ and Mn^2+^), and the C‐terminal tail with the xGxxx(V/I/L)P motif and final GR diptych is essential for catalytic activity and interaction with signaling proteins (Sun et al. 2020; Weiner and Zagzag 2000).

Structural data from isolated domains are available, but no structure of a complete LecRLK has been obtained by experimental methods to date.

### 2.8 Amaranthin‐aerolysin type lectins

Amaranthin‐aerolysin (AAT)‐like lectins are modular proteins composed of two amaranthine domains arranged in tandem in the N‐terminal region, followed by a C‐terminal domain homologous to aerolysin, a pore‐forming toxin originally described in *Aeromonas* spp. (Dang et al. 2017). Structurally, amaranthine domains exhibit a β‐trefoil fold responsible for the formation of carbohydrate‐binding sites. Each β‐trefoil domain consists of three homologous motifs arranged around a pseudo three‐fold axis to form a six‐stranded antiparallel β‐barrel capped at one end by three β‐hairpins. In classical amaranthin from *Amaranthus caudatus*, the three‐dimensional structure revealed a homodimer of approximately 66 kDa with two carbohydrate‐binding sites located at the interface between the subunits (Transue et al. 1997). This architecture is conserved in the amaranthine domains present in AAT proteins, indicating maintenance of sugar recognition potential.

Based on amaranthin, the carbohydrate‐binding site is composed of Asn74, His75, Tyr76, and Trp77 in addition to Ser124, Glu126, Ser130, and Phe135 that participate in hydrogen and van der Waals interactions with T‐antigen (Galβ1,3‐GalNAcα‐O). The N‐acetyl group of GalNAc forms a hydrogen bond with the amide of Tyr76 and a hydrophobic interaction with the aromatic ring of the same residue. The axial hydroxyl on carbon 4 of GalNAc establishes a critical interaction with the NE group of Trp77. The terminal galactose of the T‐antigen establishes hydrogen bonds, many mediated by structured water molecules, with the carbonyl groups of residues Met261, Gln263, Lys263, and Thr264 of the C‐terminal domain of the opposite subunit in addition to the side chain of Asn74 (Transue et al. 1997).

The C‐terminal domain of amaranthine‐aerolysin‐type lectins shows high similarity to bacterial aerolysin whose structure is composed mainly of β‐sheets that organize into β‐hairpins capable of forming a transmembrane β‐barrel. In typical aerolysins, this conformation allows oligomerization and insertion into target membranes, leading to pore formation (Szczesny et al. 2011). In plant‐derived AAT lectins, the structure of the aerolysin domain is conserved. However, it is still unclear whether this portion has functional cytotoxic or pore‐forming activity in plant cells. The aerolysin domain is believed to have been acquired by horizontal transfer, and its function in plants may be more related to defense mechanisms or cell signaling than to direct toxicity (Dang et al. 2017).

No experimental structure has been solved to date; only data from molecular modeling.

## 3 SYNTHETIC LECTINS AND BIOTECHNOLOGICAL APPLICATIONS

**FIGURE 5:**
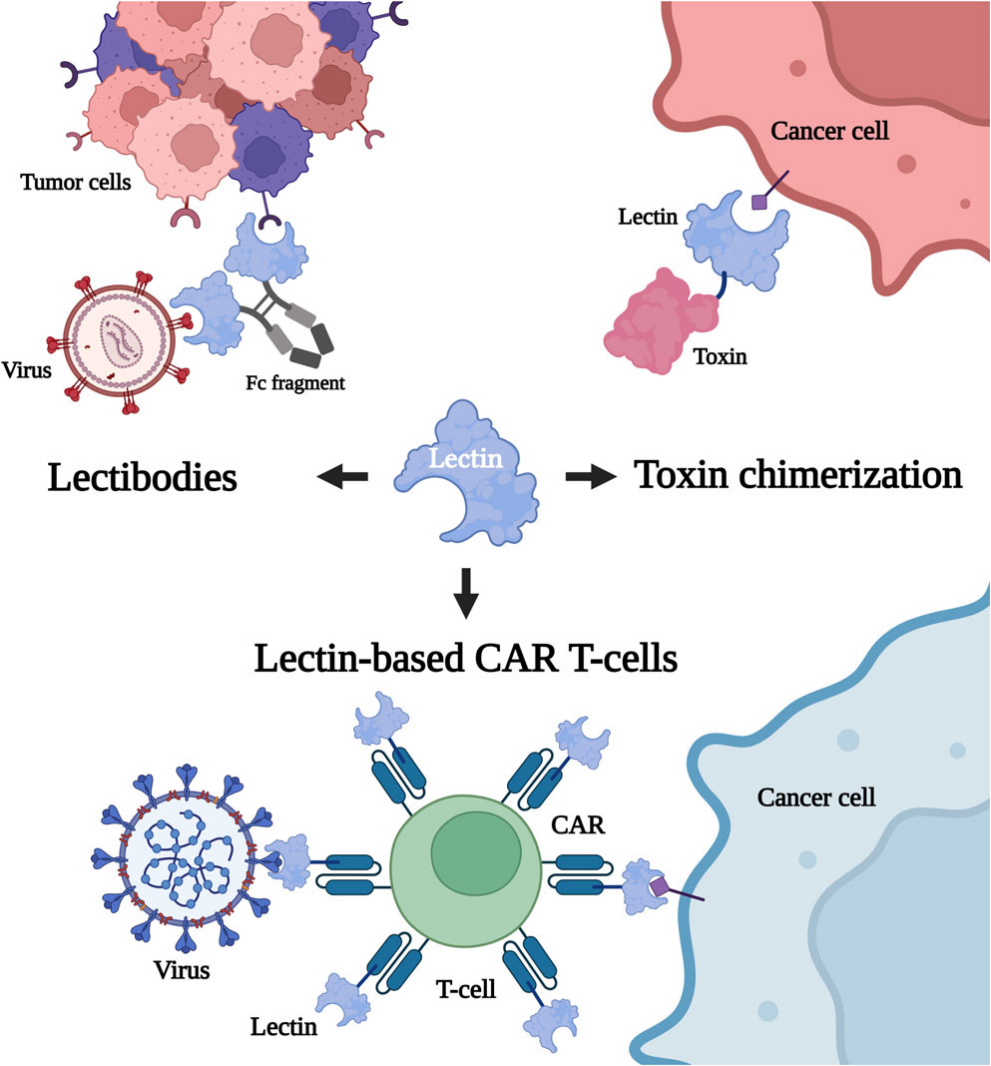
Biotechnological applications through the creation of artificial chimerolectins. Lectins can be conjugated to the Fc fragment of antibodies to generate lectibodies, bioconjugated to toxins, such as Shiga toxin, or chimerized in T cell receptors (CAR‐T) in innovative therapeutic approaches, aiming to combat viruses and cancer cells.

Based on the occurrence of natural chimerolectins and their versatility in terms of domains and functions, lectins can be combined with other functional domains, such as proteases or toxins, to generate artificial chimerolectins for biotechnological applications (Figure 5). This concept has been explored to develop therapeutic tools, mainly in the fight against cancer and viral infections (Duan et al. 2020; Notova and Imberty 2023).

Conceptually, “lectibodies” are basically chimerolectins that combine a lectin with the Fc fragment of an IgG antibody. One such chimera was developed with the actinohivin variant, a lectin specific for high‐mannose capable of recognizing HIV gp120 glycans. The lectibody showed potent antiviral activity against several HIV strains and induced death of infected cells via activation of the immune system by the Fc portion (Hamorsky et al. 2019). This strategy was also tested against cancer cells with high expression of high‐mannose N‐glycans, and it demonstrated strong binding and cytotoxic activity, reinforcing the potential of the technology (Oh et al. 2022). Combining this idea with in vitro protein binding techniques, researchers have generated libraries of lectibodies, including one based on the lectin scytovirin from the cyanobacterium *Scytonema varium*, which is also mannose‐specific (Jaakkonen et al. 2020). The lectin griffithsin (GRFT), originating from the algae *Griffithsia* sp., has the same property of interacting with HIV glycans. To enhance its action, GRFT was conjugated to the Fc region of a human antibody (IgG1), giving rise to the conjugate term mGRFT‐Fc. This modification increased its antiviral efficacy, prolonged its half‐life in the blood, and conferred the ability to activate Fc‐mediated immune responses. In addition, the mGRFT‐Fcglyc variant prevented new HIV infections by combining direct neutralization of the virus with activation of immune mechanisms (Kumariya et al. 2025). Another innovative approach was the linking of an anti‐CD3 antibody fragment to the B subunit of Shiga toxin. This chimera was able to precisely target toxicity to tumor cells expressing the Gb3 antigen (Rosato et al. 2022).

A common strategy is to fuse the lectin to toxins, targeting specific cells with altered glycosylation patterns. For example, the lectin BC2LCN from *Burkholderia cenocepacia*, which specifically recognizes H‐type 1/3 fucosylated epitopes present on human pluripotent stem cells (hPSCs), was fused to *Pseudomonas aeruginosa* exotoxin A (Tateno and Saito 2017). This chimera was shown to be effective in eliminating potentially tumorigenic hPSCs in cell cultures. This approach was also tested in pancreatic, colon, and stomach tumor cells with inhibition of tumor growth; however, the response varied among cell lines (Kitaguchi et al. 2020; Shimomura et al. 2018; Yang et al. 2022).

Additionally, lectins have been used in cell therapy strategies, such as chimeric antigen receptors (CARs) (Raglow et al. 2022). One example is the construction of a CAR based on the H84T‐BanLec, which was used to direct NK cells against SARS‐CoV‐2. This chimera demonstrated effective NK cell activation and antiviral therapeutic potential (Christodoulou et al. 2021; Swanson et al. 2015). In another study, three lectins (StxB from *Shigella dysenteriae*, LecA from *P. aeruginosa*, and a modified Mitsuba lectin) specific for α‐Gal1‐4Gal, which is present in the Gb3, were fused to CAR receptors. The resulting lectin‐based CAR‐T cells demonstrated cytotoxicity against lymphoma cell lines and also against tumor cells (Meléndez et al. 2022).

## 4 CONCLUSION

The presence of natural chimerolectins highlights the evolutionary versatility of these proteins, which perform diverse functions in organisms of different origins, from bacteria to plant, animal, and human cells. Structural studies of these lectins, including analyses of folding, domain organization, and mechanisms of action, provide valuable insights for the development of artificial chimerolectins with innovative and high‐precision biotechnological applications. These advances highlight the potential of lectins, especially in the delivery of healthcare, where they have already demonstrated promising results in the treatment of cancer and viral infections. Several classes of natural chimerolectins still lack structural data owing to the complex nature of these molecules and their subcellular location, often associated with membranes. Advances in X‐ray crystallography and electron cryomicroscopy techniques are essential for elucidating the structure of these lectins, enabling a better understanding of their functions.

## AUTHOR CONTRIBUTIONS

**Vanir Reis Pinto‐Junior:** Conceptualization; investigation; writing – original draft; writing – review and editing; validation; methodology. **Benildo Sousa Cavada:** Conceptualization; methodology; validation; investigation; funding acquisition; writing – original draft; writing – review and editing; project administration; supervision; resources. **Kyria Santiago Nascimento:** Supervision; resources; project administration; methodology; validation; writing – review and editing; writing – original draft; investigation; conceptualization; funding acquisition.

## CONFLICT OF INTEREST STATEMENT

The authors declare no conflicts of interest.

## Data Availability

The data that support the findings of this study are available from the corresponding author upon reasonable request.
